# The incidence of respiratory infections in patients undergoing major oral cavity and maxillary and mandibular resections with free flap reconstruction for head and neck cancer

**DOI:** 10.1017/S0022215125104131

**Published:** 2026-04

**Authors:** Rachel Wijayarathna, Gareth D. Jones, Samantha Ringshall, Eui-Sik Suh, Ricard Simo, Andreas Xyrichis, Nicola Peat

**Affiliations:** 1 Physiotherapy, Guy's and St Thomas' NHS Foundation Trust, London, UK; 2 King's College London, London, UK; 3 Centre for Human and Applied Physiological Sciences, King's College London, UK; 4 Lane Fox Unit, St Thomas' Hospital, London, UK; 5 Department of Otolaryngology, Head and Neck Surgery, Guy's and St Thomas' NHS Foundation Trust, London, UK

**Keywords:** Head and neck cancer, maxillofacial surgery, outcomes, primary care, respiratory medicine, cancer, head and neck surgery

## Abstract

**Objectives:**

This study aimed to analyse respiratory infection rates (RI) in a representative cohort and evaluate if tumour size, pre-existing respiratory co-morbidities, smoking history, and tracheostomy predicted postoperative infection.

**Methods:**

A retrospective observational study at a London tertiary head and neck oncology centre reviewed six years of patient data. BMJ Best Practice guidelines for hospital-acquired pneumonia (2022) were applied to medical records alongside postoperative RI prescriptions.

**Results:**

RI occurred in 32% of patients, more often in those with tracheostomy (36%) than intubation (12%). Infected patients were older (p=0.025), had tracheostomy (p=0.045), and underwent bilateral neck dissection (p<0.001). ICU (p=0.008) and hospital LOS (p<0.001) were significantly higher. Age, smoking, respiratory disease, tumour stage, and airway type were not predictors.

**Conclusion:**

RI were more frequent in tracheostomised patients, though assessed risk factors were not predictive. Further research should explore additional contributors and evaluate targeted interventions to reduce incidence.

## Introduction

Head and neck cancer (HNC) involves epithelial malignancies of the upper airway and digestive tract. These heterogeneous tumours and are the eighth leading cause of cancer in the UK.^1^ The annual incidence is 12,422 cases.^2^ Oral and oropharyngeal cancer comprises of malignant tumours located within the oral cavity and neck and rates have risen by more than 30 per cent since the 1990s.^3^ Smoking, alcohol consumption and social deprivation are important determinants and highly prevalent factors affecting the onset, prognosis and recovery from HNC.^4^ Patients often present with multiple co-morbidities making surgical management and recovery complex and high risk. Nonetheless, 45–75 per cent undergo surgery as their primary treatment.^1^ Surgery includes resection of the tumour and appropriate lymph node dissection with free flap reconstruction (FFR). Additionally, autogenous bone grafting from a secondary donor site to improve aesthetics, speech and swallow function if either the mandible or maxilla are involved.

There is a significant risk of morbidity and mortality in oral cavity cancer resections with FFR. Respiratory infections (RI) are thought to be common following surgery and is associated with an increased risk of mortality and morbidity and hospital and intensive care unit (ICU) length-of-stay (LOS).^5^ However, there is conflicting data on respiratory infection incidence rates as well as the possible predisposing factors. Small scale studies lacking statistical power and methodological rigour mean rates widely vary from 4.5 to 47 per cent. The same studies have reported tracheostomy,^6^^,^^7^ age,^8^ smoking history,^9^ respiratory co-morbidities,^10^ body mass index (BMI),^11^ anaesthetic duration,^12^ and tumour stage^13^ being associated with an increased risk of RI.

Tracheostomy insertion is common practice, 69 per cent (39/57 with FFR) and is primarily done to protect the airway during the immediate post-operative period.^14^ Weaning and decannulation of these patients is also thought to be timely and straightforward.^15^ In the acute post-operative phase, intra-oral swelling can occlude the upper airway necessitating a means of airway security.^16^^,^^17^ Timely return to theatre for flap salvage, or haemorrhage is therefore paramount. Yet intra-oral swelling can make emergency endotracheal intubation challenging.

The mechanisms by which patients undergoing this major surgery can develop an RI are numerous. Pre-operative health and fitness can impact post-operative complications, while prolonged anaesthetic times increase the risk of RI.^18^ Alterations in respiratory mechanics can predispose patients to RI. Resecting and denervation of structures involved with swallowing and airway protection increases the risk of aspiration. A tracheostomy bypasses the body’s normal filtration and humidification systems, drying the lining of the airways and secretions. Glottic closure is absent reducing cough effectiveness. The requirement for regular suction can also introduce pathogens into the airways, increasing the risk of RI. What’s more, multiple attachments and reduced space in the ICU can restrict mobility as does pain from multiple surgical sites. Restrictions in movement to protect microvascular reconstructions further contribute to this risk.

Current literature lacks a clear definition of the prevalence and risk factors for RI. Understanding this prevalence would enable better risk stratification, allowing for targeted interventions that could reduce hospital and ICU stays, as well as the associated care costs of major surgery.

## Materials and methods

The primary aim of the study is to describe and analyse the rates of RI in patients who underwent oral cavity, maxillary and mandibular cancer resections with FFR. The secondary aim was to analyse whether tumour size, pre-existing respiratory co-morbidity and smoking history influenced RI rates.

### Design

This was a retrospective observational study conducted over a 1-month period from 31 October 2022 at a specialist centre for head and neck surgical oncology at a single centre tertiary London hospital.

Homogenous convenience sampling was employed to capture cohorts of patients who underwent both methods of airway security. Data between 2018 and 2021 were collected to capture patients who underwent tracheostomy. Prior to this, there was a wide variation in case selection, meaning some patients underwent overnight intubation. Data from a 2-year period (2014–2015) captured patients who underwent overnight intubation. The design of the study did not require a sample size calculation.

Eligible participants were identified from a database collected as part of routine clinical service evaluation and audit. Paper and electronic medical records were screened and retrospective data extracted. Eligibility criteria included all patients who underwent oral cavity cancer, maxillary and mandibular resections with FFR. Patients who underwent surgery during the previous 12 months, and those who underwent salvage surgery or surgery for osteoradionecrosis, were excluded.

### Ethical approval

The study received HRA approval (number 22/HRA/3386) and local Research and Development (R&D) sponsorship in accordance with the Code of Ethics of the World Medical Association (Declaration of Helsinki; 2013). The retrospective design precluded need of consent. However, in order to comply with National Data Op-out (NDOO) legislation, the NDOO database was screened and patients who selected opt-out were excluded.

### Outcome measure

The primary outcome was evidence of RI and was recorded using a dual approach. The “British Medical Journal (BMJ), best practice guidelines on hospital-acquired pneumonia (non–Covid-19)”^19^ (2022) was retrospectively applied to medical records between post-operative days 1 and 7.

A new and/or persistent shadowing (consolidation) on chest x-ray, which was otherwise unexplained, plus at least two of the following confirmed the diagnosis: fever greater than 38°C (>100ºF), leukocytosis (WBC >10 × 10^9^/L) or leucopenia (WBC <4 × 10^9^/L), purulent sputum and decline in oxygenation.

The number of post-operative reactive antibiotic prescriptions for chest infection during the same post-operative period was recorded from prescription charts. If patients scored positively consolidation plus two or more of the same four variables, the presence of RI was recorded. If they were absent despite antibiotic prescription, RI was not recorded.

All patients received similar post-operative care and physiotherapy input, centred around preventing post-operative pulmonary complications. This included regular nebulisation with saline and mucolytics, early mobility, regular analgesia and strategies to ensure regular effective secretion clearance. Antibiotic prescription both post-operatively (Co-amoxiclav for 3 days) and for RI was standardised as per local guidelines.

### Data collection

A single primary investigator, a specialist physiotherapist for head and neck cancer surgery, undertook the data collection, recording and analysis. Descriptive demographic data including age, sex, smoking history, respiratory history, Eastern Cooperative Oncology Group Performance Status (ECOG-PS), tumour site and stage were collected. Data relating to the surgery type, date, the type and levels of neck dissection and flap site were also recorded. Temporal measures included date return to theatre, ICU and overall hospital LOS.

### Data analysis

Descriptive statistics are presented as frequencies and percentages for categorical variables and mean (±SD) for continuous variables. Continuous data were not normally distributed (Kolmogorov–Smirnov, age *p* = 0.004, pack-year history *p* < 0.001, ICU LOS *p* < 0.001, hospital LOS *p* = 0.001). Therefore, Mann–Whitney *U* rank-based tests were used to assess differences in age, pack-year history, ICU and hospital length of stay between patients who did and did not develop an RI).

A chi-squared test of homogeneity was used to assess proportional differences in categorical variables between those who developed an RI and those who did not. For cells with an expected cell count of below 5, Fisher’s exact test was used instead (chronic obstructive pulmonary disease [COPD], asthma, sarcoidosis, readmission to critical care and return to surgery). Binomial logistic regression was used to predict the risk of a patient developing a respiratory infection with respect to airway type, age, respiratory history, smoking history and tumour stage. All data were analysed using IBM Statistical Package from the Social Sciences (SPSS) Version-25 with statistical significance assumed if *p* was less than or equal to 0.05. The Strengthening the Reporting of Observational Studies in Epidemiology (STROBE) guidelines were followed to report this study (appendix 1).

## Results

A total of 127 patients were identified (mean age 62.1 (±11.7) years) of which 80 (63 per cent) were male (Table 1). A total of 110 patients had a tracheostomy, and 17 were intubated. Mean ICU LOS was 2(±2) days, and hospital LOS was 17(±12) days (Table 2). Most patients (70 per cent) had multiple areas resected, with 97 per cent undergoing neck dissection. Only 4 per cent required ICU readmission, and 7 per cent returned to theatre, 5 per cent for flap issues, one needing two return trips for flap salvage and another three times.

**Table 1.** Patient demographics

Patient demographics		n=127
Age (Mean ±)		62.1 ± 11.7
Gender	**Female %**	37 (n=47)
	**Male %**	63 (n=80)
Smoking history	**Never %**	41 (n=52)
	**Ex-smoker %**	35 (n=44)
	**Current %**	24 (n=31)
Pack year history (Mean: SD)		14 ± 21
Missing pack year history (Mean ±)		16 (n)
Respiratory History %		22 (n=28)
COPD %		11 (n=14)
Asthma %		9 (n=12)
Sarcoidosis %		1 (n=1)
PS %	**0**	78 (n=99
	**1**	13 (n=16)
	**2**	8 (n=10)
	**Missing**	2 (n)
Tumour stage %	**Stage 1**	17 (n=22)
	**Stage 2**	32 (n=41)
	**Stage 3**	5 (n=6)
	**Stage 4**	46 (n=58)

**Table 2.** Surgical demographics of included patients

Surgical demographics		N = 127
Airway %	**Intubation**	13 (*n* = 17)
	**Tracheostomy**	87 (*n* = 110)
Number of sites resected %	**1**	71 (*n* = 90)
	**2**	25 (*n* = 32)
	**3**	4 (*n* = 5)
Maxillectomy %		19 (*n* = 24)
Mandibulectomy %		42 (*n* = 53)
Glossectomy %		35 (*n* = 44)
Floor of mouth %		17 (*n* = 21)
Palate %		2 (*n* = 3)
Buccal mucosa %		13 (*n* = 16)
Retromolar trigone %		2 (*n* = 3)
Orbit %		1 (*n* = 1)
Neck dissection %	**Neck dissection**	97 (*n* = 123)
	**Unilateral**	80 (*n* = 101)
	**Bi-lateral**	17 (*n* = 22)
	**Selective neck dissection**	95 (*n* = 121)
	**Modified radical**	2 (*n* = 2)
	**Radical**	0
	**Level 1−2**	2 (*n* = 3)
	**Level 2−4**	2 (*n* = 2)
	**Level 1−3**	32 (*n* = 41)
	**Level 1−4**	54 (*n* = 69)
	**Missing (levels)**	7 (n)
Flap type %	**Radial forearm**	40 (*n* = 51)
	**Antero-lateral thigh**	16 (*n* = 20)
	**Fibula**	21 (*n* = 27)
	**Pectoralis major**	3 (*n* = 4)
	**Scapula**	15 (*n* = 19)
	**Medial sural artery perforator**	4 (*n* = 5)
	**Latissimus dorsi**	1 (*n* = 1)
	**Serratus anterior**	1
Length of ICU stay (Mean ±)		2 ± 2
Readmission to critical care %		4 (*n* = 5)
Return to surgery %		7 (*n* = 9)
Reason %	**Wound debridement**	1 (*n* = 1)
	**Debulk flap**	2 (*n* = 3)
	**Washout**	1 (*n* = 1)
	**Evacuation of haematoma and redo flap**	1 (*n* = 1)
	**flap salvage**	1 (*n* = 1)
	**Neck swelling**	1 (*n* = 1)
	**Trachy change in theatre**	1 (*n* = 1)
Respiratory infection %		32 (*n* = 41)
Hospital LOS (Mean ±)		17 ± 12

RI was identified in *n* = 41 (32 per cent) patients. Age was statistically significantly higher in those with a RI (median rank 65.5) than those without (median rank 63.0), [*U* = 2223, z = 2.246; *p* = 0.25] using an exact sampling distribution for U.^20^ Hospital LOS was statistically significantly higher in those with a RI (median rank 18.5) than those without (median rank 11.00), [*U* = 2702, z = 4.706, *p* < 0.001]. ICU LOS was statistically significantly higher in those with a RI (median rank 2.0) than those without (median rank 1.0), [*U* =2271, z = 2.661, *p* = 0.008] (Table 3).

**Table 3.** Characteristics of included patients who developed a respiratory infection compared to those without

Respiratory Infection		No infection	Infection	*p*-Value
		*N* = 86	*N* = 41	
Airway %	**Intubation**	88 (*n* = 15)	12 (*n* = 2)	0.045*
	**Tracheostomy**	64 (*n* = 70)	36 (*n* = 40)	
Age (Mean ±)		61±11.9	65±10.8	0.025*
Gender %	**Female**	24 (*n* = 31)	13 (*n* = 16)	0.858
	**Male**	43 (*n* = 54)	20 (*n* = 26)	
Smoking history %	**Non-Smoker**	28 (*n* = 36)	13 (*n* = 16)	0.646
	**Smoking history**	39 (*n* = 49)	33 (*n* = 26)	
pack year history (Mean ±)		11±19	19±24	0.84
Respiratory history %		13 (*n* = 16)	19 (*n* = 12)	0.213
	**COPD**	5 (*n* = 6)	6 (*n* = 8)	0.067
	**Asthma**	6 (*n* = 8)	3 (*n* = 4)	1.000
	**Sarcoidosis**	1 (*n* = 1)	0 (*n* = 0)	1.000
PS %	**0**	49 (*n* = 68)	24 (*n* = 31)	0.851
	**1 & 2**	19 (*n* = 17)	7 (*n* = 9)	
Tumour stage %	**1 & 2**	36 (*n* = 46)	13 (*n* = 17)	0.148
	**3 & 4**	31 (*n* = 39)	20 (*n* = 25)	
Number of areas resected intra-orally	**One**	67 (*n* = 62)	33 (*n* = 31)	0.157
	**More than one**	68 (*n* = 23)	32 (*n* = 11)	0.917
Neck dissection type %	**Neck dissection**	67 (*n* = 83)	33 (*n* = 40)	0.257
	**Unilateral**	59 (*n* = 75)	20 (*n* = 26)	<0.001*
	**Bi-lateral**	6 (*n* = 8)	11 (*n* = 14)	
length of ICU stay (Mean ±)		2 ± 1	3 ± 3	0.008*
Readmission to critical care %		2 (*n* = 2)	2 (*n* = 3)	0.326
Return to surgery %		5 (*n* = 6)	2 (*n* = 3)	1.000
Hospital LOS (Mean ±)		14 ± 8	24 ± 14	<0.001*

* Denotes statistical significance < 0.05.

There was a statistically significant higher rate of RI in those who had a tracheostomy (36 per cent) compared to those who were intubated (12 per cent), a difference in proportions of 0.24 [χ^2^(df) = x0.000; *p* = 0.45]. There was also a statistically significant higher rate of RI in those that had a bilateral neck dissection (64 per cent) compared with a unilateral neck dissection (27 per cent), a difference of proportions of 0.37 [χ^2^(df) = x0.000; *p* < 0.001]. There were no other statistically significant differences in proportions between all other variables.

The logistic regression models for age (*p* = 0.050), smoking history (*p* = 0.881), respiratory history (*p* = 0.246), tumour stage (*p* = 0.142) and airway type (*p* = 0.064) were not statistically significant.

## Discussion

The study observed a 32 per cent incidence rate of RI, this is higher than the 12 per cent and 15 per cent reported in the existing literature.^21^^,^^22^ A study used Health Information Service (HIS) data to identify pneumonia and the Melbourne Group Scale (MGS) to identify post-operative pulmonary complications (PPCs), in a similar group of patients.^22^ The authors reported a 12 per cent and 15 per cent incidence rate respectively. However, small samples and methodological flaws make comparison to the current data difficult, and true prevalence ambiguous. Rates could be under-reported as prospective data, standardised outcomes were not utilised^21^ and financial incentives associated with HIS data could lead to over-reporting.^22^ The MGS identified all PPC’s meaning data specific to RI could not extracted. The current study was conducted at a tertiary specialist centre for HNC surgery, handling complex surgery on high-risk patients, which may explain the higher infection rates. However, the lack of detailed patient and surgical demographics prevented direct comparison with the current study.^21^^,^^22^

Post-operatively, the alterations in respiratory mechanics and pathophysiology do lend themselves to the high incidence rates described. This combined with prevalence demonstrates that this is a clinical issue that needs to be addressed. The surgical insult to structures vital to breathing and management of secretions strongly supports the narrative that tracheostomy insertion is necessary.^23^^–^^25^ This, combined with the high prevalence of tracheostomy insertion in UK head and neck surgical cancer centres, demonstrates this will not change.^15^ Therefore, identifying those patients most at risk and directing interventions to improve outcome is necessary.

We observed a higher proportion of older patients and patients undergoing bilateral neck dissections who developed an RI. These variables have not been identified in the very limited existing literature. However, age has been identified as a significant influencing factor on post-operative morbidity and mortality in other surgical specialities.^26^ Despite a higher proportion of older patients, and patients undergoing tracheostomy insertion who developed an RI, logistic regression analysis did not identify them as predictors. Logistic regression modelling requires a minimum of 15 cases per independent variable, some report this should be higher than 50.^27^ These minimum case numbers were not met for both logistic regression models, likely accounting for the non-significant results. The study was of retrospective design; therefore, a power calculation was not performed, and we should acknowledge that the study may have been under-powered to make accurate predictions.

Critical care and hospital LOS was significantly longer in patients who developed a respiratory infection. Similar findings were described in a study reporting on the incidence of tracheostomy related complications in HNC surgery.^28^ In patients who developed a complication, hospital LOS increased from 14 to 25 days.^28^ Presentation of the data did not allow for a separate analysis of only those that developed an RI. However, the significant increases in ICU and hospital LOS for patients that develop an RI support the notion that patients who develop complications have a longer hospital LOS.

There is a plethora of evidence demonstrating the efficacy of respiratory physiotherapy and early mobility in reducing post-operative pulmonary complications, morbidity and LOS.^29^^–^^33^ Although evidence is largely confined to critical care and other surgical specialties, these positive outcomes may be translatable to the head and neck surgical population. Implementing standardised physiotherapy pathways, combined with early risk stratification to identify high-risk patients (e.g., those with pre-existing conditions), could enhance recovery and reduce morbidity, mortality and LOS in patients who undergo major head and neck surgery. This evidence gap combined with the high rates of RI seen in the current study highlights the need for future studies to investigate the efficacy, timing and dosing of targeted respiratory physiotherapy and early mobility in post-operative major head and neck surgery and how they may reduce complication rates.

- Oral cavity, maxillary and mandibular cancer rates are increasing and so is the number of patients undergoing curative major surgery
- Oral cavity, maxillary and mandibular surgery is complex and high risk, as is the population that undergo this type of surgery
- While morbidity and mortality are high, little evidence exists on peri-operative risk factors and the incidence on respiratory infection
- The findings of this study demonstrate a clinically significant incidence rate of respiratory infection in patients who undergo major oral cavity resections with free flap reconstruction
- The findings of this study demonstrate the complexity of these patients, identifying a significantly longer ICU and hospital LOS in those who develop an infection
- This study investigates certain pre- and peri-operative risk factors that may predict the onset of an infection

## Supporting information

10.1017/S0022215125104131.sm001 Wijayarathna et al. supplementary material Wijayarathna et al. supplementary material

## Supplementary material

The supplementary material for this article can be found at https://doi.org/10.1017/S0022215125104131.

## References

[1] Ferlay J, Shin H-R, Bray F, Forman D, Mathers C, Parkin DM. Estimates of worldwide burden of cancer in 2008: GLOBOCAN 2008. *Int J Cancer* 2010;127:2893–917 21351269 10.1002/ijc.25516

[2] Cancer Research UK Head and neck cancer statistics. 2015–2017. In: Head and neck cancers statistics | Cancer Research UK [5 July 2023]

[3] Office for Cancer Information and Utilisation (OCIU). Profiles of head and neck cancers in England: incidence, mortality and survival 2010 [5 July 2023]. http://www.ociu.nhs.uk/sph-documents/Head_and_Neck_Profiles.pdf

[4] McCarter K, Baker AL, Britton B, Wolfenden L, Wratten C, Bauer J, et al. Smoking, drinking, and depression: comorbidity in head and neck cancer patients undergoing radiotherapy. *Cancer Med* 2018;7:2382–90 29671955 10.1002/cam4.1497 PMC6010893

[5] Graves N, Weinhold D, Tong E, Birrell F, Doidge S, Ramritu P, et al. Effect of healthcare-acquired infection on length of hospital stay and cost. *Infect Control Hosp Epidemiol* 2007;28:280–92 17326018 10.1086/512642

[6] Coyle MJ, Tyrrell R, Godden A, Hughes CW, Perkins C, Thomas S, et al. Replacing tracheostomy with overnight intubation to manage the airway in head and neck oncology patients: towards an improved recovery. *Br J Oral Maxillofac Surg* 2013;51:493–6 23929589 10.1016/j.bjoms.2013.01.005

[7] Crosher R, Baldie C, Mitchell R. Selective use of tracheostomy in surgery for head and neck cancer: an audit. *Br J Oral Maxillofac Surg* 1997;35:43–5 9043003 10.1016/s0266-4356(97)90008-5

[8] Loeffelbein DJ, Julinek A, Wolff K-D, Kochs E, Haller B, Haseneder R. Perioperative risk factors for postoperative pulmonary complications after major oral and maxillofacial surgery with microvascular reconstruction: a retrospective analysis of 648 cases. *J Craniomaxillofac Surg* 2016;44:952–7 27259678 10.1016/j.jcms.2016.05.007

[9] Rao MK, Reilley TE, Schuller DE, Young DC. Analysis of risk factors for postoperative pulmonary complications in head and neck surgery. *Laryngoscope* 1992;102:45–7 1731156 10.1288/00005537-199201000-00008

[10] Petrar S, Bartlett C, Hart RD, MacDougall P. Pulmonary complications after major head and neck surgery: a retrospective cohort study. *Laryngoscope* 2012;122:1057–61 22447296 10.1002/lary.23228

[11] Jones NF, Jarrahy R, Song JI, Kaufman MR, Markowitz B. Postoperative medical complications—not microsurgical complications—negatively influence the morbidity, mortality, and true costs after microsurgical reconstruction for head and neck cancer. *Plast Reconstr Surg* 2007;119:2053–60 17519700 10.1097/01.prs.0000260591.82762.b5

[12] Patel RS, McCluskey SA, Goldstein DP, Minkovich L, Irish JC, Brown DH, et al. Clinicopathologic and therapeutic risk factors for perioperative complications and prolonged hospital stay in free flap reconstruction of the head and neck. *Head Neck* 2010;32:1345–53 20091687 10.1002/hed.21331

[13] Semenov YR, Starmer HM, Gourin CG. The effect of pneumonia on short-term outcomes and cost of care after head and neck cancer surgery. *Laryngoscope* 2012;122:1994–2004 22777881 10.1002/lary.23446

[14] Marsh M, Elliott S, Anand R, Brennan PA. Early postoperative care for free flap head and neck reconstructive surgery—a national survey of practice. *Br J Oral Maxillofac Surg* 2009;47:182–5 18640751 10.1016/j.bjoms.2008.06.004

[15] Cameron M, Corner A, Diba A, Hankins M. Development of a tracheostomy scoring system to guide airway management after major head and neck surgery. *Int J Oral Maxillofac Surg* 2009;38:846–9 19423295 10.1016/j.ijom.2009.03.713

[16] Coyle MJ, Shrimpton A, Perkins C, Fasanmade A, Godden D. First do no harm: should routine tracheostomy after oral and maxillofacial oncological operations be abandoned. *Br J Oral Maxillofac Surg* 2012;50:732–5 22325994 10.1016/j.bjoms.2012.01.003

[17] Goetz C, Burian NM, Weitz J, Wolff KD, Bissinger O. Temporary tracheotomy in microvascular reconstruction in maxillofacial surgery: benefit or threat. *J Craniomaxillofac Surg* 2019;47:642–6 30755353 10.1016/j.jcms.2019.01.017

[18] Farwell DG, Reilly DF, Weymuller EA Jr, Greenberg DL, Staiger TO, Futran NA. Predictors of perioperative complications in head and neck patients. *Arch Otolaryngol Head Neck Surg* 2002;128:505–11 12003580 10.1001/archotol.128.5.505

[19] BMJ Best Practice. *Management of Hospital-Acquired Pneumonia (non-COVID-19)*. In Hospital-acquired pneumonia (non COVID-19) - Symptoms, diagnosis and treatment | BMJ Best Practice US [4 February 2022]

[20] Dineen LC, Blakesley BC. Algorithm AS 62: a generator for the sampling distribution of the Mann-Whitney U statistic. *Appl Stat* 1973;22:269–73

[21] Littlewood CG, Jebril A, Lowe D, Konig R, Groom P, Rogers SN. Factors contributing to delayed decannulation of temporary tracheostomies following free tissue reconstructive surgery for head and neck cancer. *Br J Oral Maxillofac Surg* 2021;59:472–7 33485712 10.1016/j.bjoms.2020.09.019

[22] Shaw LM, Iseli TA, Wiesenfeld D, Ramakrishnan A, Granger CL. Postoperative pulmonary complications following major head and neck cancer surgery. *Int J Oral Maxillofac Surg* 2021;50:302–8 32682644 10.1016/j.ijom.2020.06.011

[23] Bianchi B, Copelli C, Ferrari S, Ferri A, Sesenna E. Free flaps: outcomes and complications in head and neck reconstructions. *J Craniomaxillofac Surg* 2009;37:438–42 19553132 10.1016/j.jcms.2009.05.003

[24] Singh T, Sankla P, Smith G. Tracheostomy or delayed extubation after maxillofacial free-flap reconstruction. *Br J Oral Maxillofac Surg* 2016;54:878–82 27287181 10.1016/j.bjoms.2016.05.026

[25] Zubair F, McMahon J, Carson E, McCaul J, Hislop WS, Wales C, et al. Unscheduled return to the operating theatre after head and neck surgery with free flap repair. *Br J Oral Maxillofac Surg* 2022;60:554–7

[26] Thompson DA, Makary MA, Dorman T, Pronovost PJ. Clinical and economic outcomes of hospital-acquired pneumonia in intra-abdominal surgery patients. *Ann Surg* 2006;243:547–52 16552208 10.1097/01.sla.0000207097.38963.3b PMC1448956

[27] Menard S. *Applied Logistic Regression Analysis 2nd edn*. Thousand Oaks: Sage Publications, 2002

[28] Castling B, Telfer M, Avery BS. Complications of tracheostomy in major head and neck cancer surgery: a retrospective study of 60 consecutive cases. *Br J Oral Maxillofac Surg* 1994;32:3–5 8136336 10.1016/0266-4356(94)90162-7

[29] Morris PE, Goad A, Thompson C, Taylor K, Harry B, Passmore L, et al. Early intensive care unit mobility therapy in the treatment of acute respiratory failure. *Crit Care Med* 2008;36:2238–43 18596631 10.1097/CCM.0b013e318180b90e

[30] Boden I, Skinner EH, Browning L, Reeve J, Anderson L, Hill C, et al. Preoperative physiotherapy for the prevention of postoperative pulmonary complications: systematic review and meta-analysis. *BMJ* 2018;360:j5916 29367198 10.1136/bmj.j5916 PMC5782401

[31] Svensson-Raskh A, Schandl AR, Ståle A, Nygren-Bonnier M, Olsén MF. Mobilization started within 2 hours after abdominal surgery improves peripheral and arterial oxygenation: a randomized controlled trial. *Phys Ther* 2021;101:pzab094 33742678 10.1093/ptj/pzab094 PMC8136304

[32] Afxonidis G, Kokkoris S, Papadopoulos C, Nikolaou C, Mouloudi E, Koukoulitsios I, et al. Efficacy of early and enhanced respiratory physiotherapy in ICU patients: a meta-analysis. *PM Open Respir Care* 2021;16:870–9

[33] Tazreean R, Tan J, Lewis C, Campbell JP. Early mobilization in enhanced recovery after surgery: a systematic review. *Perfusion* 2022;37:588–96

